# Evolutionary Analysis of Infectious Bronchitis Virus Reveals Marked Genetic Diversity and Recombination Events

**DOI:** 10.3390/genes11060605

**Published:** 2020-05-29

**Authors:** Mohammed A. Rohaim, Rania F. El Naggar, Mohammed A. Abdelsabour, Mahmoud H. A. Mohamed, Ibrahim M. El-Sabagh, Muhammad Munir

**Affiliations:** 1 Department of Virology, Faculty of Veterinary Medicine, Cairo University, Giza 12211, Egypt; mohammed_abdelmohsen@cu.edu.eg (M.A.R.); ibrahimelsabagh@yahoo.com (I.M.E.-S.); 2 Division of Biomedical and Life Sciences, Faculty of Health and Medicine, Lancaster University, Lancaster LA1 4YG, UK; 3 Department of Virology, Faculty of Veterinary Medicine, University of Sadat City, Sadat 32897, Egypt; rania.elnagar@vet.usc.edu.eg; 4 Department of Poultry Viral Vaccines, Veterinary Serum and Vaccine Research Institute (VSVRI), Agriculture Research Centre (ARC), Cairo 11381, Egypt; dr.m.adel.vsvri@gmail.com; 5 Department of Clinical Sciences, College of Veterinary Medicine, King Faisal University, P.O. Box 400, Al-Ahsa 31982, Saudi Arabia; mahmoudhassanain@yahoo.com; 6 Department of Avian and Rabbit Medicine, College of Veterinary Medicine, Zagazig University, Zagazig 44511, Egypt; 7 Central Biotechnology Laboratory, College of Veterinary Medicine, King Faisal University, P.O. Box 400, Al-Ahsa 31982, Saudi Arabia

**Keywords:** infectious bronchitis virus, Saudi Arabia, selective pressure, recombination, evolution, vaccines

## Abstract

In the last 5 years, frequent outbreaks of infectious bronchitis virus (IBV) are observed in both broiler and layer chicken flocks in the Kingdom of Saudi Arabia (KSA) in spite of extensive usage of vaccines. The IBV is a widespread avian coronavirus affecting both vaccinated and unvaccinated chicken flocks and is attributed to significant economic losses, around the globe. In the present study, 58 (*n* = 58) samples were collected from four different commercial poultry flocks from 8 KSA districts during 2019. A total of nine positive isolates (9/58; 15.5%), based on real-time reverse transcriptase PCR targeting nucleocapsid (N) gene, were used for further genetic characterization and evolutionary analysis. Genetic characterization of the partial spike (S1) gene revealed the clustering of the reported isolates into three different genotypes, whereas four additional isolates were grouped within 4/91 genotype, two isolates within IS/885 genotype, one isolate was closely related to IS/1494/06, and two isolates were grouped within classic serotype (vaccine-like strains). Phylodynamic revealed clustering of four isolated viruses within GI-13 lineage, three isolates within GI-23 lineage, and two isolates within GI-1 lineage. Results indicate that there are high evolutionary distances between the newly identified IBV strains in this study and the commercially used vaccines (GI-1), suggesting that IBV strains circulating in the KSA are under constant evolutionary pressures. Selective pressure biostatistics analyses consistently demonstrate the presence of a higher positive score which highlights the role of natural selection, a mechanism of virus evolution on sites located on the protein surface, within or nearby domains involved in viral attachment or related functions. Recombination analysis revealed emergence of two isolates through recombination events resulting in new recombinant viruses. Taken together, these finding demonstrate the genetic and evolutionary insights into the currently circulating IBV genotypes in KSA, which could help to better understand the origin, spread, and evolution of infectious bronchitis viruses, and to ascertain the importance of disease monitoring as well as re-evaluation for the currently used vaccines and vaccination programs.

## 1. Introduction

Infectious bronchitis virus (IBV) is a highly contagious and emerging viral disease-causing organism of chickens and belongs to genus *Gammacoronavirus* within the family Coronaviridae [1]. Infectious bronchitis virus (IBV) can replicate efficiently in a wide variety of epithelial cells of respiratory, renal, reproductive, and digestive tracts [1]. IBV is an enveloped, single-stranded, and positive-sense RNA virus with genome length of approximately 27.6 kb which encodes four major structural proteins, spike (S), membrane (M), envelope (E), phosphorylated nucleocapsid (N) proteins, and several accessory proteins (3a, 3b, 5a, and 5b) [2,3]. The spike protein consists of two subunits: S1 and S2. The S1 forms the extracellular part of virus and plays a major role in tissue tropism, induction of protective immunity, virus neutralization, cell attachment, and serotype specificity, whereas S2 subunit anchors the spike into the virus membrane. During IBV replication and evolution, the high mutation rate in the S1 gene generates substantial genotypical, antigenic, and pathogenic variations. The existing infectious bronchitis vaccines primarily failed to provide cross-protection against these multiple genotypes and serotypes [4,5]. Owing to decisive roles of S1 in immunity and virus diversity, the IBV genetic classification and evolutionary analysis are mainly based on the S1 gene sequences [6,7,8,9,10,11,12].

Continuous evolution of IBV variants in various regions remains a major concern for chicken production, around the world [13]. Till now, vaccination is considered to be the most effective control approach against IBV; however, current vaccines have been found to be ineffective due to the continuous emergence of newly evolving viruses [14,15]. Multiple IBV serotypes, genotypes, and pathotypes have been identified worldwide since its first description in 1931 in America [12,16]. Recent classification of IBV has identified seven main genotypes (GI–GVII), 35 distinct lineages (1−35), and a number of inter-lineage recombinants based on the sequencing analysis of the entire S1 gene of IBV strains isolated from different countries, around the worldwide [17,18,19]. Despite mass vaccination strategies in Saudi Arabia utilizing Mass-type (H120, M41, and Ma5) and 793B-type (CR88 and 4/91) of vaccine, variant IBVs are still devastating the poultry industry. Similarly, IB poses a significant economic impact on poultry industry of Saudi Arabia. The aim of the present study is to genetically characterize field IBV strains and determine the genetic divergence between these field circulating strains and the currently used vaccines in Saudi Arabia. These findings guide the selection and engineering of appropriate vaccine candidates to effectively curtail the IB infection in the country.

## 2. Materials and Methods

### 2.1. Ethical Statement

The clinical samples used in this study were collected in strict accordance with the guidelines for Animal Ethics Committees, King Faisal University, Saudi Arabia. Samples of tracheas were collected from diseased and/or dead chickens, stored at −80 °C till used, for diagnosis and virus isolation. The diseased chickens examined in this study were received at Department of Clinical Studies, College of Veterinary Medicine, King Faisal University, Al Hofuf, Saudi Arabia and approved by the poultry farm owners. Diagnosis and experimental protocols in this study were approved by College of Veterinary Medicine, King Faisal University Animal Care Committee.

### 2.2. Clinical Information, Samples Collection, and Processing

During 2019, 58 samples were collected from different chicken flocks in 4 Saudi Arabian districts (i.e., Buradiya, Al Hofuf, Dammam, and Al Duwadimi). The collected samples were originated from poultry flocks experiencing clinical forms of the disease as respiratory symptoms or problems with egg production associated with high mortality and morbidity rates. Epidemiological information of the studied isolates is summarized in Table 1. Tracheas collected from diseased flocks showed congestion along with caseous exudates, cloudiness, and turbidity of air sacs. Furthermore, degeneration of the ovary and swollen oviducts associated with egg peritonitis was observed in layer flocks. Virus isolation and propagation was carried out through inoculation of the tissue homogenate of pooled samples (10 pooled samples) from each farm into the allantoic cavity of specific-pathogen-free embryonated chicken eggs (SPF ECEs) (9–11-day-old) [20]. Inoculated eggs were monitored daily by candling for embryonic mortality. Two days after inoculation, the allantoic fluid was harvested and further passed into another three successive passages with 2 days duration for each passage.

### 2.3. Viral RNA Extraction and Genetic Characterization

RNA was extracted directly from the allantoid fluid using a QIAamp Viral RNA Mini Kit (Qiagen, Valencia, CA, USA) according to the manufacturer’s instructions. Detection of IBV was conducted using a real-time reverse transcriptase PCR based on the highly conserved N gene, as described previously [21]. For spike (S1) gene amplification, RNA was reverse transcribed into DNA using a Superscript IV First-Strand cDNA Synthesis Kit (Invitrogen, Waltham, MA, USA) and the second strand was synthesized with the addition of Q5 DNA Polymerase (New England Biolabs, Ipswich, MA, USA). The RT-PCRs were conducted using One Step RT-PCR Kit (Qiagen, Hilden, Germany) according to the manufacturer’s instructions using previously published primers for amplification and sequencing of the whole S1 coding region [2,3,4,5,6,7,8,9,10,11,12,13,14,15,16,17,18,19,20,21,22,23,24,25]. Amplified PCR products were visualized by electrophoresis on a 1.5% agarose gel electrophoresis and then purified using a QIAquick Gel Extraction Kit (Qiagen, Hilden, Germany) following the manufacturer’s instructions.

### 2.4. S1 Coding Region Sequencing, Sequence Analysis, and Phylogeny

To further characterize the biology and evolution of the newly identified IBV strains, full-length S1 gene sequencing was carried out using BigDye Terminator v3.1 Sequencing Kit (Applied Biosystems, Foster City, CA, USA) and an automated sequencer (ABI, 3500, Applied Biosystems, Foster City, CA, USA). The quality of obtained S1 gene sequences were checked, assembled, and edited using BioEdit software version 7.0.4.1 [26] and submitted to GenBank using BankIt tool of the GenBank (http://www.ncbi.nlm.nih.gov/WebSub/?tool=genbank), and the accession numbers were obtained.

Phylogenetic analysis was conducted to explore the phylogenetic relationships with high-level of clustering pattern dependent on the full-length S1 gene between the reported isolates and recently described strains from the Middle East including Saudi Arabia and other parts of the world. Alignments of the obtained nucleotide sequences of full-length S1 genes were performed using the Multiple Alignment using Fast Fourier Transform (MAFFT) method in Geneious software, v11.1.3 (Biomatters, Auckland, New Zealand). The alignments were then exported to the MEGA program, v7.0.26 [27]. Maximum likelihood (ML) phylogenetic analyses of the S1 coding region were then conducted using the best-fitting nucleotide substitution models (the lowest Bayesian information criterion (BIC) scores in each analysis were for the general time reversible (GTR) model and a discrete gamma distribution (+G) with five rate categories, assuming that a certain fraction of sites are evolutionarily invariable (G + I)). Bootstrap analyses of the resultant trees were performed using 1000 replicates. Trees were finally visualized and annotated using FigTree v1.4.2 software (http://tree.bio.ed.ac.uk/software/figtree/).

### 2.5. Antigenicity Prediction and 3D Visualization of Mutations

Previous studies have reported that most of the neutralizing epitopes and receptor-binding domain of S1 protein were distributed in the three hypervariable regions (HVRs), including HVR I (38 aa–67 aa), HVR II (91 aa–141 aa), and HVR III (274 aa–387 aa), which were usually employed to classify IBV genotypes [28,29]. Deduced amino acid sequences alignment of the S1 subunit of the S protein was performed by comparing with the parental viruses. To further investigate the HVRs and mutations in the newly identified IBV strains, the 3D structure of IBV/CH/SA/1/2019 isolate and IBV/CH/SA/6/2019 S1 proteins were constructed by homology modelling method. From the PDB database, the glycoprotein S1 structure was downloaded and used as the template for modelling S1 monomer. All the HVRs and mutations of representative isolates from each lineage were identified and visualized by PyMOL software (http://www.pymol.org/).

### 2.6. Evolutionary Analysis

Sequence alignments were conducted, in barrel with different available viruses resembling different genotypes; GI-1, GI-13, and GI-23. The RDP4 software (v4.97) was used to detect any recombination events in the obtained sequences [30]. The S1 coding region sequences of the isolated strains in this study were screened in barrel with different available viruses resembling genotypes GI-1, GI-13, and GI-23 to check if unusual clusters formed by Saudi Arabian IBV strains are viruses representing new recombinant IBV. The RDP4 analysis was accomplished using different available methods with their default parameters; however, recombination events were only considered proven if detected by at least seven algorithms (RDP, Geneconv, BootScan, MaxChi, Chimaera, SiScan, and 3Seq) and the *p*-value was calculated below 1.0 × 10^−30^. Selective pressure for the putative S1 protein was predicted using synonymous nonsynonymous analysis program (SNAP) services (http://hcv.lanl.gov/content/sequence/SNAP/SNAP.html). The ratio of nonsynonymous to synonymous substitutions (dN/dS) for each amino acid site in the S1 coding region was used to scan for evidence of positive or negative selection.

## 3. Results

### 3.1. Virus Detection and Molecular Epidemiology of Infectious Bronchitis Virus

We present the isolation and integrative genetic analysis that map the evolution of IBV in Saudi Arabia during 2019. Typical IBV lesions such as embryo curling and dwarfism were detected in embryonated chicken eggs (ECEs) after three successive passages. Using real-time RT-PCR assay that targets the N gene, only nine samples were detected positive (9/58) (Table 1). Furthermore, there were notable variations in the proportion of positive samples between four districts. The lowest detection rates were observed in Al Duwadimi (1/11; 1.2%), and Al Hofuf (2/16; 12.5%) and Dammam (2/13; 15.4%) were at the mid-point, whereas the highest rates were detected at Buradiya (4/18; 22.2%) (Table 1).

### 3.2. Phylogenetic Analysis

**Figure 1:**
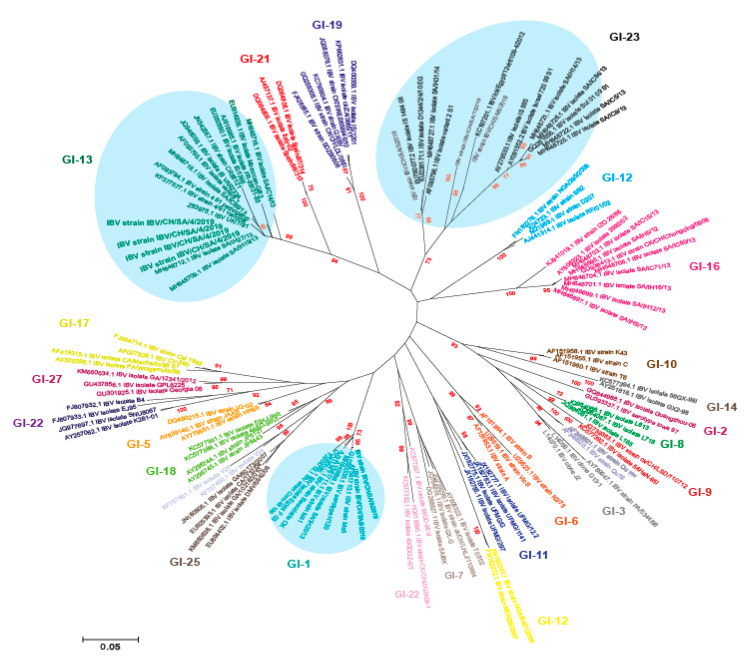
Phylogenetic analysis based on S1 gene of the newly identified infectious bronchitis viruses (IBVs) in Saudi Arabia during 2019 showed the clustering pattern for the studied isolates with different IBV lineages labelled with circle.

**Figure 2:**
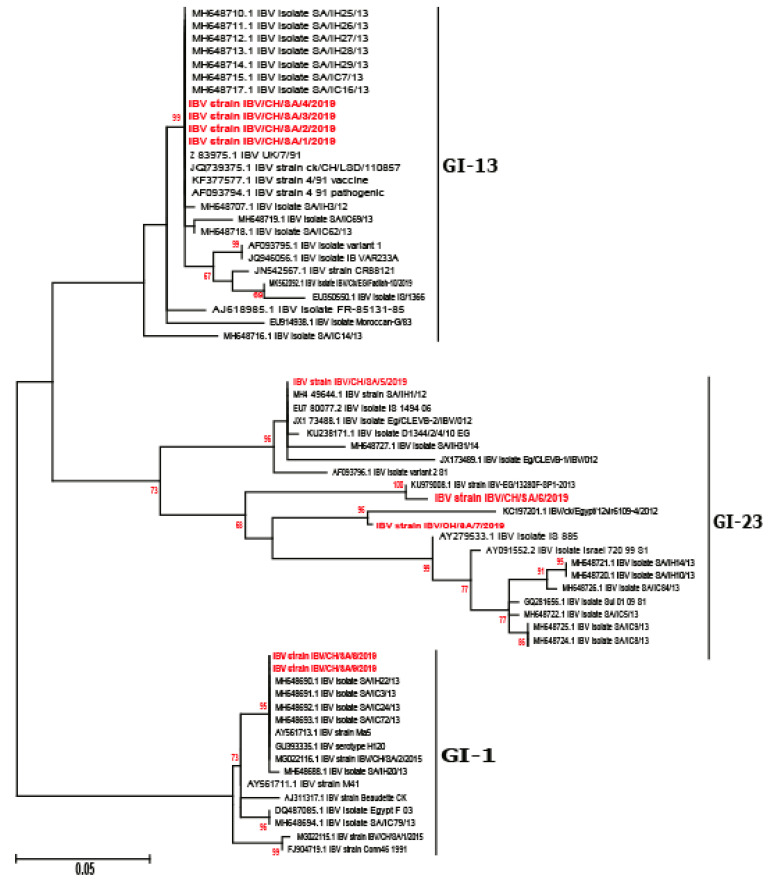
A higher resolution phylogenetic tree for GI-1, GI-13, and GI-23 lineages; the reported isolates are marked with red color. The robustness of individual nodes of the tree was assessed using 1000 replications of bootstrap resampling of the originally aligned nucleotide sequences. The scale bar represents the number of substitutions per site. The year of isolation and geographical origin of the virus sequences are included in the tree.

Phylogenetic relationship among IBV strains has been established based on the analysis of the full-length S1 gene. Previous studies for virus characterization are based on the sequence of either HVR I-II or the HVR-III regions only. However, S1 gene sequences including three HVRs should be carried out for both differentiation between the vaccine and field strains and for accurate genotyping [6,7,8]. Our results indicated that Saudi Arabian IBV viruses in this study were diversified into three distinct genotypes, i.e., GI-1, GI-13, and GI-23 (Figure 1 and Figure 2) according to Valsastro et al. [17]. Multiple reports have demonstrated the emergence of variant strains related to 4/91, IS/1494, and IS/885 (IS/720/99) within the Middle East [31,32]. A Bayesian consensus phylogenetic analysis, which was verified using the neighbor-joining method, clearly divided isolated IBV strains in this study into three lineages. A total of nine reported isolates were clustered with isolates of lineage GI-1, GI-13, and GI-23 in association with previously reported strains from Saudi Arabia and Middle East (Figure 2).

### 3.3. Comparative Amino Acid Analysis between Currently Circulating IBV Strains and Locally Used Vaccines

The isolated IBV strains were genotyped based on sequencing and subsequent sequence analysis for the S1 gene. The obtained nucleotide and corresponding amino acid sequences were aligned and compared with parental origin and vaccine strains. Sequences of the IBV strains detected in this study were submitted to the GenBank database and the following accession numbers MT270486–MT270494 were assigned. The characterized viruses in this study shared different levels of nucleotide and amino acid sequence identities compared to currently used vaccines in Saudi Arabia, which were between 75% and 90%. Out of the nine reported isolates, two isolates (SA/8/2019 and SA/9/2019) were closely related to GI-1 (vaccine strain) and four identified isolates ((SA/1/2019, SA/2/2019, SA/3/2019, and SA/4/2019) were closely related to 4/91 genotype. These isolates showed distinctive amino acids mutations in the HVRs compared to their parental origin and CR88 vaccine strain, which is commonly used in Saudi Arabian poultry (Table 2). Likewise, three isolates (SA/5/2019, SA/6/2019, and SA/7/2019) were found closely related to IS/720/99 and revealed mutations within all HVRs (Table 3).

**Figure 3:**
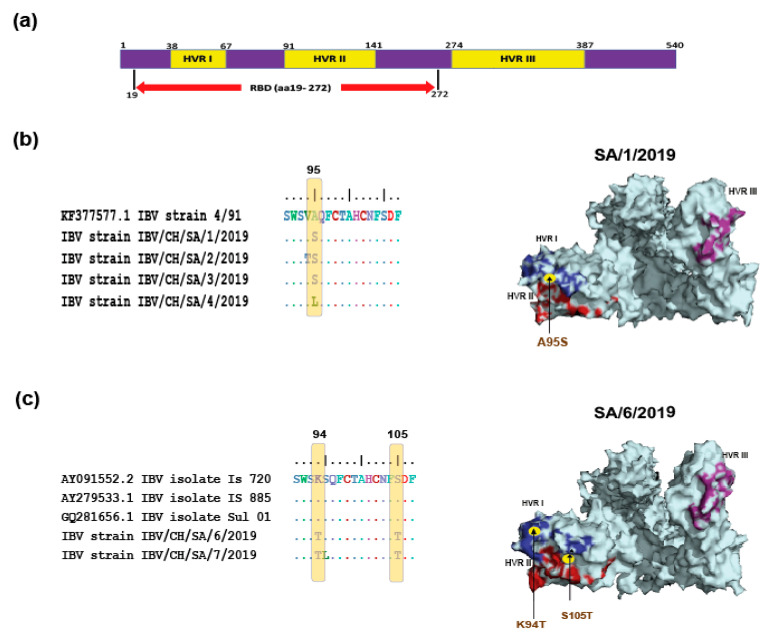
Characterization and localization of specific mutations in the spike protein of the newly identified IBV strains in comparison with their parental origin (GI-1, GI-13, and GI-23 lineages). (**a**) Schematic diagram based on the identified functional domains of S1 protein. (**b**) The multi-alignment of S1 glycoprotein, conducted by BioEdit, showed the location of specific mutation sites within the receptor-binding domain (RBD). Briefly, 3D structure template was downloaded from PDB database, and the hypervariable regions (HVR) regions of IBVs SA/1/2019 (**b**) and SA/6/2019 (**c**) isolates compared to their parental origin 4/91 (793b) genotype and IS/720/99 genotypes, respectively, were predicted. The 3D structure was visualized by PyMOL software.

Compared with the 3D capsid structure of reported isolates in the PDB database (PDB ID: 6CV0), prominent amino acid substitutions were located in the major receptor-binding domain (aa 19–aa 253) (Figure 3a) and adjacent to HVR I and HVR II regions. Specifically, IBV/CH/SA/1/2019, IBV/CH/SA/2/2019, and IBV/CH/SA/3/2019 isolates showed substitutions at residues A95S, while IBV/CH/SA/4/2019 isolate carried A95L compared to their parental 4/91 genotype (793/B) (Figure 3b). On the other hands, IBV/CH/SA/6/2019 and IBV/CH/SA/7/2019 showed substitutions at residues K94T and S105T, respectively, compared to their parental origin IS/720/99 (Figure 3c). Given the roles of these residues in virus neutralization, these mutations might change the virus antigenicity and facilitate the emergence of variant strains.

**Figure 4:**
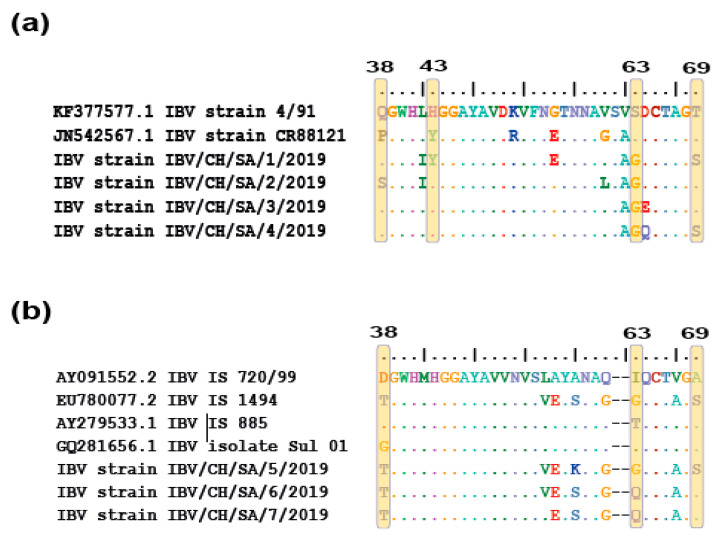
The multi-alignment of residue substitutions associated with virus tropism. (**a**) IBV isolates closely related to 4/91 genotype showed mutations at positions 38, 43, 63, and 69 in comparison with their parental origin (GI-13). (**b**) IBV isolates closely related to IS/720/99 showed mutations at positions 38, 63, and 69 in comparison with their parental origin (GI-23 lineage).

Moreover, residues at positions 38, 43, 63, and 69 are critical for binding of the IBV spike protein to the receptors in the chicken respiratory tract [3]. In the analyzed sequences of the isolated viruses, residue substitutions were identified for four isolates genetically related to 4/91 genotype: SA/2/2019 isolate at position 38, SA/1/2019 isolate at position 43, and SA/1/2019 and SA/4/2019 isolates at position 69. All four isolates have substitutions at reside 63 compared to their parental origin 4/91 (Figure 4a). On the other hand, three isolates closely related to IS/720/99 have substitutions at position 38 and 63, while SA//2019 isolate has single mutation at position 69 compared to their parental origin IS/720/99 (Figure 4b).

### 3.4. Recombination Analysis and Selective Pressure

**Figure 5:**
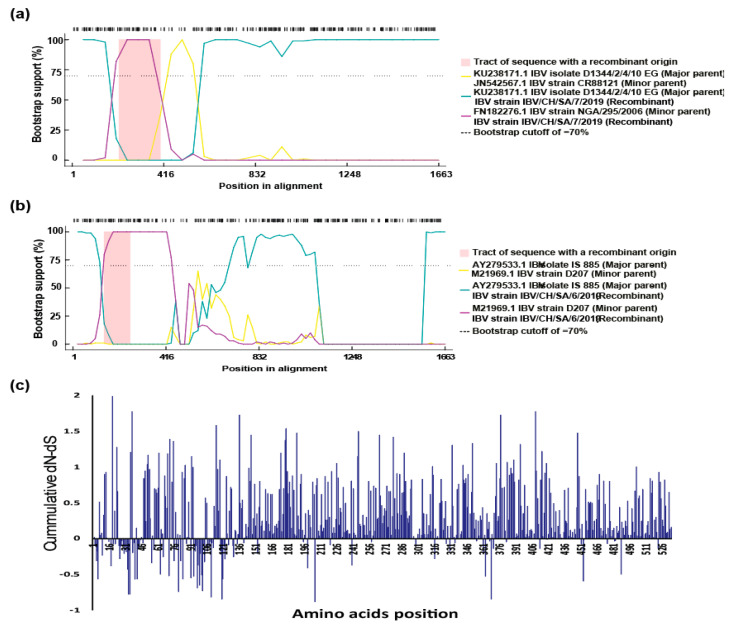
Recombination and selective pressure analysis. Recombination detection analysis displaying possible recombination events predicted within the S1 segment of the (**a**) IBV strain IBV/CH/SA/7/2019 isolate and (**b**) IBV strain IBV/CH/SA/6/2019. **(c)** Cumulative difference between the nonsynonymous substitution rate and the synonymous substitution rate (dN-dS) using SNAP methods along S1 protein of the IBV strains sequenced in this study.

As a highly variable coronavirus, numerous IBV variants have been identified and nucleotide substitutions or recombination between field strains and vaccines occurred frequently. We further investigated the possible recombination events within our isolated viruses and other IBV genotypes using the RDP4 software. The results showed that two recombinant events resulting in emergence of two new recombinant viruses, which are detected in the current study with high score *p* < 0.01 and recombinant score >0.6. The recombinant IBV strain IBV/CH/SA/7/2019 could be as a result of recombination between two strains, major parent is belonging to GI-23 lineage, while the minor parent is closely related to GI-13 lineage, which is a vaccine like strain (CR88) (Figure 5a), whereas IBV strain IBV/CH/SA/6/2019 probably emerged from two different lineages of GI-12 (minor parent) and GI-23 (major parent) (Figure 5b). To further evaluate the presence of a differential selective pressure strength on the S1 gene, the standardized differences in dN−dS were calculated for each position. Scores higher than 0 are suggestive of a more prominent diversifying selection (Figure 5c). Additionally, this value was used to calculate a cumulative score by summing it codon-by-codon. This score allowed highlighting the diversification tendency of S1 protein regions. Our results demonstrated that the cumulative difference between the nonsynonymous substitution rate (dN) and the synonymous substitution rate (dS) (i.e., dN-dS) for the Saudi Arabian IBV strains were under high positive selection along the spike (S1) protein (Figure 5c).

## 4. Discussion

Notoriously, IBV strains can undergo many genetic modifications generated both by recombination and mutations such as substitutions, deletions, and insertions, which could lead to the emergence of new variants. There are characteristic features of coronaviruses in the genome structure (large single-stranded RNA) and virus biology (minimal proofreading activity of viral polymerase) and immunological pressure caused by the vaccines that create favorable conditions for such mutation events [33]. It is well known that even point mutation in the amino acid sequence of the spike protein can result in emergence of novel genotypes and/or serotypes that might be antigenically different from the existing classic and variant vaccine strains [27,28]. Previous studies have shown that changes as little as 5% in the S1 gene can alter protective efficacy of the vaccines [33].

Currently, several new genotypes or serotypes have been reported worldwide as a result of mutations within the S1 gene [17,18,19]. A new classification and nomenclature system, based on the whole S1 gene, has been proposed, which categorizes all IBV strains into 35 lineages and 7 genotypes (GI to GVII) [17,18,19]. IBV was first reported in Saudi Arabia in 1984 [34]. Several IBV lineages were previously reported in Saudi Arabia, including GI-13 or 4/91, GI-16 or CK/CH/LDL/97I, GI-1 or Mass IBV, and GI-23 or Middle East IBV, which were subdivided into two subgroups, i.e., IS/720/99 and IS/Variant2/98 [34,35,36,37,38]. All these studies indicated that IBV strains circulating in Saudi Arabia are genetically diverse, providing a potential platform for recombination and greatly challenging the current biological control measures.

In this study, we focused on the molecular characterization of IBVs within the Saudi Arabian commercial poultry flocks. Genotyping was accomplished by phylogenetic analysis of the S1 coding region sequences against reference strains representing all genotypes and lineages [17,18,19] followed by recombination analysis. In spite of the antigenic relatedness between currently circulating genotypes (GI-1, GI-13, and GI-23 lineages) in Saudi Arabia, the comparative analysis with vaccine strains of Massachusetts-type (Ma5) strains and others strains types (e.g., 793/B serotype) has not been conducted. Vaccination represents the most effective strategy to limit economic losses of IBV. However, higher antigenic variability and low cross-protection between the circulating genotypes require the usage of different vaccine combinations in order to broaden the protection spectrum [10,39]. Therefore, the development of broadly cross-reactive vaccines against recently emerged genotypes is urgently warranted. Unfortunately, even closely related vaccines can fall into episodes of incomplete protection or vaccine immune escape because of amino acid substitutions in specific antigenic sites [10,39].

Phylogenetic analysis indicated that IBV strains in this study were of three distinct genotypes: GI-1, GI-13, and G-23 [17,18,19]. Only two detected viruses, i.e., IBV/CH/SA/8/2019 and IBV/CH/SA/9/2019, were similar to Ma5 strains and were grouped with GI-1 lineage. However, the pathogenicity of these strains needs to be further investigated as these viruses were isolated from clinically infected chickens with high morbidity and mortality rates.

Hypervariable regions (HVRs) in the S1 gene showed distinct patterns with prominent amino acid substitutions among different viruses compared to the parental origin for the isolated viruses in this study. Some of these amino acid residues are responsible for the virus tropism and pathogenesis at positions 38 (T/Q/S), 43 (Y), 63 (G/Q), and 69 (S/A) in the receptor-binding site. Likewise, using the protein 3D modelling prediction, there are three amino acid substitutions near the HVR I/II, which might help in the virus evolution leading to change in the antigenicity and pathobiology especially due to localization of these mutations within the RBD.

IBV of GI-13, GI-16, and GI-23 lineages have been circulating in the Middle East region for the previous 20 years and are also detected in Saudi Arabia [34,35,36,37,38]. The phylogenetic analyses performed on the full-length S1 genes of nine IBV strains isolated in this study showed that they cluster into different groups, i.e., GI-1, GI-13, and GI-23 lineages. These subsequent IBV lineages could have reached to Saudi Arabian poultry because of international trade, including uncontrolled movement of animals across borders [39,40] or through the virus carried by wild birds from the neighboring countries including Egypt [32]. Likewise, strains similar to those circulating in Egypt, India, China, and Italy have also been previously reported in Saudi Arabia [34,35,36,37,38].

The role of vaccination in driving viral evolution has been reported for different diseases affecting both animals and human beings [41]. When immunity is not sterilizing, wild bird strains are able to circulate in a new and more challenging environment [42,43]. Selective pressure analysis illustrated that the circulating Saudi Arabian IBVs are under high pressure, suggesting the role of vaccination in virus evolution along with virus adaptation in wild birds. The recombination events were detected in at least two strains of IBV; however, one of them (IBV/CH/SA/7/2019) is emerging due to recombination between GI-23 lineage and CR88 vaccine strain (GI-13 lineage). Many recombination events have been reported in different IBV strains, not only between field (wild type) and vaccine viruses but also among field viruses either within the same genotype (intragenotypic) or between different genotypes (intergenotypic), leading to emergence of new genotypes [42,43]. The employed recombination detection methods revealed that IBV/CH/SA/6/2019 and IBV/CH/SA/7/2019 strains have undergone intergenotypic recombination and different breakpoints within the S1 gene, which might be a precursor for further IBV evolution in Saudi Arabia in the near future. A potential issue attributed to the recombination is that changes occurring during recombination in the S1 subunit might alter the neutralizing epitopes and result in the appearance of novel IBV serotypes. In addition, recombination events between vaccine and field strains may lead to reversal of virulence and genesis of highly virulent strains [44]. Therefore, further studies are required to clearly determine the clinicopathobiological features of these viruses in a chicken model. Interestingly, the GI-23 genotype plays a two-faced role in these recombinant events in the Saudi Arabian poultry flocks, not only providing fragments for other strains but also receiving fragments during infection, which has been reported previously in neighboring countries [31,32].

## 5. Conclusions

In conclusion, continuous monitoring and surveillance for IBVs is required not only in commercial poultry flocks but also in wild birds which will elucidate sequence characteristics of prevailing strains to help revising and updating the vaccination strategies to develop and or choose a vaccine that can give cross-protection against majority of the circulating genotypes. Transmission of IBVs through wild birds has been reported in neighboring countries to Saudi Arabia such as Israel, Jordan, and Egypt, highlighting that wild birds might play a vital role in the virus epidemiology. The current study revealed the cocirculation of multiple IBV genotypes within Saudi Arabia owing to high mutation rate and subsequent residue substations leading to the evolution of IBV variants and potentially, vaccination failure. Likewise, further studies are required to understand the genetic and biological characteristics of IBV variants in relation to antigenicity, pathogenicity, tropism, and shedding as well as evolution-driven failure in vaccine efficacy.

## Figures and Tables

**Table 1:** Sampling data and prevalence of infectious bronchitis viruses (IBVs) in four geographical regions in Saudi Arabia.

Sampling Site	Sampled (*n*)	Breed	Positive (*n*)	Rate (%)
Buradiya	7	Broilers	1	22.2
	11	Layers	3	
Al Hofuf	10	Broilers	1	12.5
	6	Layers	1	
Dammam	8	Broilers	2	15.4
	5	Layers	0	
Al Duwadimi	5	Broilers	0	9
	6	Layers	1	

**Table 2:** Sequence alignment of hypervariable regions (HVRs) amino acid sequences for four isolates in this study compare to their parental origin 4/91 genotype (793/B serotype).

Strain	HVR1 (60–88)	HVR2 (115–140)	HVR3 (275–292)
**4/91**	VSVSDCTAGTFYESYNISAASVAMTVPPA	FKSQQGSCPLTGMIPQNHIRISAMRS	TFTNVSNASPNSGGVDTF
**CR88**	**G. A . . . . . . . . . . . H. . . . S . . . . . . HN**	**. . N . L . . . . . . . . . . . . . . . . . . . . D**	**S . . . . . . . . . . . . . . . . .**
**SA/1/2019**	**. . AG . . . . . S . . . . K . . . . . . . . . . . . . .**	**. . . . . . . . . . . . . . . . . . . . . . . . . Y**	**. . . . . . . . . . . . . . . . . .**
**SA/2/2019**	**. . AG . . . . . . . . . . . . F . . . . . . . . A . . .**	**. . . . . . . . . . . L . . . . . . . . . . . . . .**	**. . . . . . . . . . . . . . . . . .**
**SA/3/2019**	**. . AGE . . . . . . . . . . . F . . S . . . . . A . . .**	**. . . . . . . . . . . . . . . . . . . . . . . . . Y**	**. . . . . . . . . . . . . . . . . .**
**SA/4/2019**	**. . AGQ . . . . S. . . . . . . . . . . . . . . . . .**	**. . . . . . . . . . . . . . . . . . . . . . . . . .**	**. . . . . . . . . . . . . . . . . .**

A dot indicates an identical amino acid.

**Table 3:** Sequence alignment of HVRs amino acid sequences for three reported isolates in this study compared to their parental origin IS/720/99.

Strain	HVR1 (60–88)	HVR2 (115–140)	HVR3 (275–292)
**IS/720/99**	Q- - IQCTVGAIGWSKNFSAASVAITAPAA	YSSGQGSCPLTGQLQRNSIRISAMSG	TFYNESNAPPNVGGVNTI
**IS/1494/06**	**G. . GQ . . A . S . Y . . . . . . . S . . . M . . . DT**	**. K . . H . . . . . . . LIPQ . H . . . . . . KN**	**. . T . V . . . S . . T . . . . . .**
**SA/5/2019**	**G . . GQ . . A . S . Y . . . . . T . S . . . M . . . DT**	**. KN . . . . . . . . . LIPQ . H . . . . . . KN**	**. . T . V . . . S . . T . . . . . .**
**SA/6/2019**	**G . . QQ . . A . . . Y . . . . . . . . . . . M . . . QN**	**. K . SS . . . . . . . MIPQYY . . . . . . RN**	**. . H . . . . . H . . N . . . H . .**
**SA/7/2019**	**G . . QQ . . A . . . Y . . . . . . . . . . . M . . . QN**	**. K . SS . . . . . . . MIPQHY . . . . . . RN**	**. . . . . . . . S . . S . . . . . .**

A dot indicates an identical amino acid. A dash indicates an amino acid deletion.
